# Protocatechuic acid as a potent anticarcinogenic compound in purple rice bran against diethylnitrosamine-initiated rat hepatocarcinogenesis

**DOI:** 10.1038/s41598-022-14888-2

**Published:** 2022-06-22

**Authors:** Charatda Punvittayagul, Theerapat Luangsuphabool, Rawiwan Wongpoomchai

**Affiliations:** 1 Research Affairs, Faculty of Veterinary Medicine, Chiang Mai University, Chiang Mai, 50100 Thailand; 2 Biotechnology Research and Development Office, Department of Agriculture, Rangsit, Thanyaburi, Pathum Thani 12110 Thailand; 3 Department of Biochemistry, Faculty of Medicine, Chiang Mai University, Chiang Mai, 50200 Thailand; 4 Research Center of Producing and Development of Products and Innovations for Animal Health and Production, Faculty of Veterinary Medicine, Chiang Mai University, Chiang Mai, 50100 Thailand

**Keywords:** Biochemistry, Cancer

## Abstract

Our previous study demonstrated that purple rice bran extract (PRBE) could inhibit diethylnitrosamine (DEN)-induced hepatocarcinogenesis. Protocatechuic acid (PCA) is the major phenolic acid contained in the PRBE. Therefore, this study aimed to determine whether PCA is an anticarcinogenic compound in purple rice extract. Rats were intraperitoneally injected with DEN to induce glutathione *S*-transferase placental form (GST-P)-positive foci. Rats were fed with PRBE at 500 mg kg^−1^ body weight or PCA at 4 mg kg^−1^ body weight for 5 and 15 weeks. PCA administration attenuated DEN-induced hepatic GST-P positive foci to a degree similar to PRBE. The molecular mechanisms of PCA in the initiation stage were correlated with reduced activity of cytochrome P450 reductase and induction of glutathione *S*-transferase. In addition, PCA also downregulated the expression of *TNF-α* and *IL-1β* genes in rat liver. These genes are associated with the inhibition of inflammation. In the promotion stage, PCA suppressed cell proliferation correlated with the downregulation of *Cyclin D1* expression. Moreover, it also induced apoptosis, indicated by increased expression of *P53* and *Bad* genes, and decreased the expression of the anti-apoptotic *Bcl-xl* in DEN-initiated rats. These findings suggest that PCA is an active compound in the anticarcinogenic action of purple rice bran.

## Introduction

Globally, liver cancer is the third leading cause of cancer death^1^. The major risk factors are associated with hepatitis B and hepatitis C virus infection, fatty liver disease, alcohol consumption, and aflatoxin B_1_. There are several treatment options to cure cancer, such as surgical resection, liver transplantation, chemotherapy, immunotherapy, and oncolytic virus therapy^2^. However, the survival rate of most patients with liver cancer is low due to late diagnosis, drug resistance, and cancer recurrence, resulting in a poor prognosis^3^.

There is now an increasing number of scientific reports to support the use of foods and food components as cancer therapeutic candidates. Food-derived bioactive compounds could prevent all stages of carcinogenesis via diverse pathways, such as antioxidant and detoxifying enzyme systems, cell proliferation and death, cell cycle, and the inflammatory response^4,5^. For example, allicin, the main organic allyl sulfur compound in garlic, can inhibit the growth of cholangiocarcinoma tumor in a HuCCT-1 xenograft nude mouse model^6^. Apigenin, a flavonoid found in many fruits and vegetables, can attenuate the growth of human chondrosarcoma tumor in a nude mouse model by decreasing cell proliferation and increasing cell apoptosis^7^. Vanillic acid, a phenolic acid found in many plant species such as rice, rye, and almond skin, can prevent rat hepatocarcinogenesis induced by diethylnitrosamine and 1,2-dimethylhydrazine^8^. Ellagic acid, a phenolic acid found in berries, nuts, and pomegranates, can suppress cell proliferation and induce apoptosis in MCF-7 and MDA-MB-231 breast cancer cells^9^.

Rice (*Oryza sativa* L.) is an important source of nutrition, especially in Asia. Rice bran, the outer layer of the rice grain, contains phytochemical compounds which possess several types of biological activity such as antioxidant, anti-inflammatory, hepatoprotective, and anticancer activity^10–13^. Our previous study demonstrated that purple rice bran extract has higher anticancer activity against diethylnitrosamine-induced rat hepatocarcinogenesis than white rice bran. Based on chemical analysis, we found that protocatechuic acid is a major phenolic acid in purple rice extract^14^. Therefore, this study aimed to investigate whether protocatechuic acid is an anticarcinogenic compound in purple rice extract. In addition, the inhibitory mechanism of protocatechuic acid against diethylnitrosamine-induced rat hepatocarcinogenesis was also evaluated.

## Materials and methods

### Chemicals

Diethylnitrosamine (DEN) and 3, 3’-diaminobenzidine tetrahydrochloride hydrate were obtained from Sigma-Aldrich (St. Louis, MO, USA). ApopTag Peroxidase in situ kit was purchased from Merck (Darmstadt, Germany). Rabbit polyclonal antibody to rat glutathione *S*-transferase placental form (GST-P) was purchased from MBL (Nagoya, Japan). Protocatechuic acid (PCA) was purchased from Alfa-Aesar (Karlsruhe, Germany). Vectastain ABC kit was purchased from Vector Laboratories, Inc. (Burlingame, CA, USA). EnVision Doublestain system was purchased from Dako (Glostrup, Denmark). All other chemicals were analytical grade.

### Purple rice bran extraction

Purple rice (*Oryza sativa* L. var. *indica*) cv. Kum Doi Saket bran was obtained from the Faculty of Agriculture, Chiang Mai University, Thailand, having been cultivated from August to November 2016. A voucher specimen, Rawiwan-001, has been deposited in the archives of the Faculty of Pharmacy (Herbarium number: CMU; 023,252), Chiang Mai University, Thailand. Briefly, the purple rice bran was twice defatted by hexane and then extracted twice with absolute methanol for 48 h at room temperature. After filtration, the solution was evaporated and freeze-dried to obtain the purple rice bran extract (PRBE). The extract was then kept at − 20 °C until further use.

### Animals

Male Wistar rats (4 weeks old) were purchased from the National Laboratory Animal Center, Mahidol University, Salaya, Nakorn-Prathom, Thailand. Rats were housed in groups of three per cage under a 12 h light/dark cycle and 50–60% humidity at 23 ± 2 °C in the Animal House, Faculty of Medicine, Chiang Mai University, Chiang Mai, Thailand. Feed and tap water were provided ad libitum. All procedures were approved by The Animal Ethics Committee of the Faculty of Medicine, Chiang Mai University (Protocol No. 20/2560). All methods were performed in accordance with the relevant guidelines and regulations. This study follows the recommendation in the ARRIVE guidelines.

### Experimental protocol

Our previous experiment indicated that PRBE can inhibit the DEN-induced early stage of rat hepatocarcinogenesis^14^. Therefore, to find out the active compounds contained in the extract, PCA, the most abundant phenolic compound in the extract, was selected for this study. The concentration of 4 mg kg^−1^ body weight (BW) of PCA was equivalent to PCA content found in 500 mg kg^−1^ BW of PRBE.

**Figure 1:**
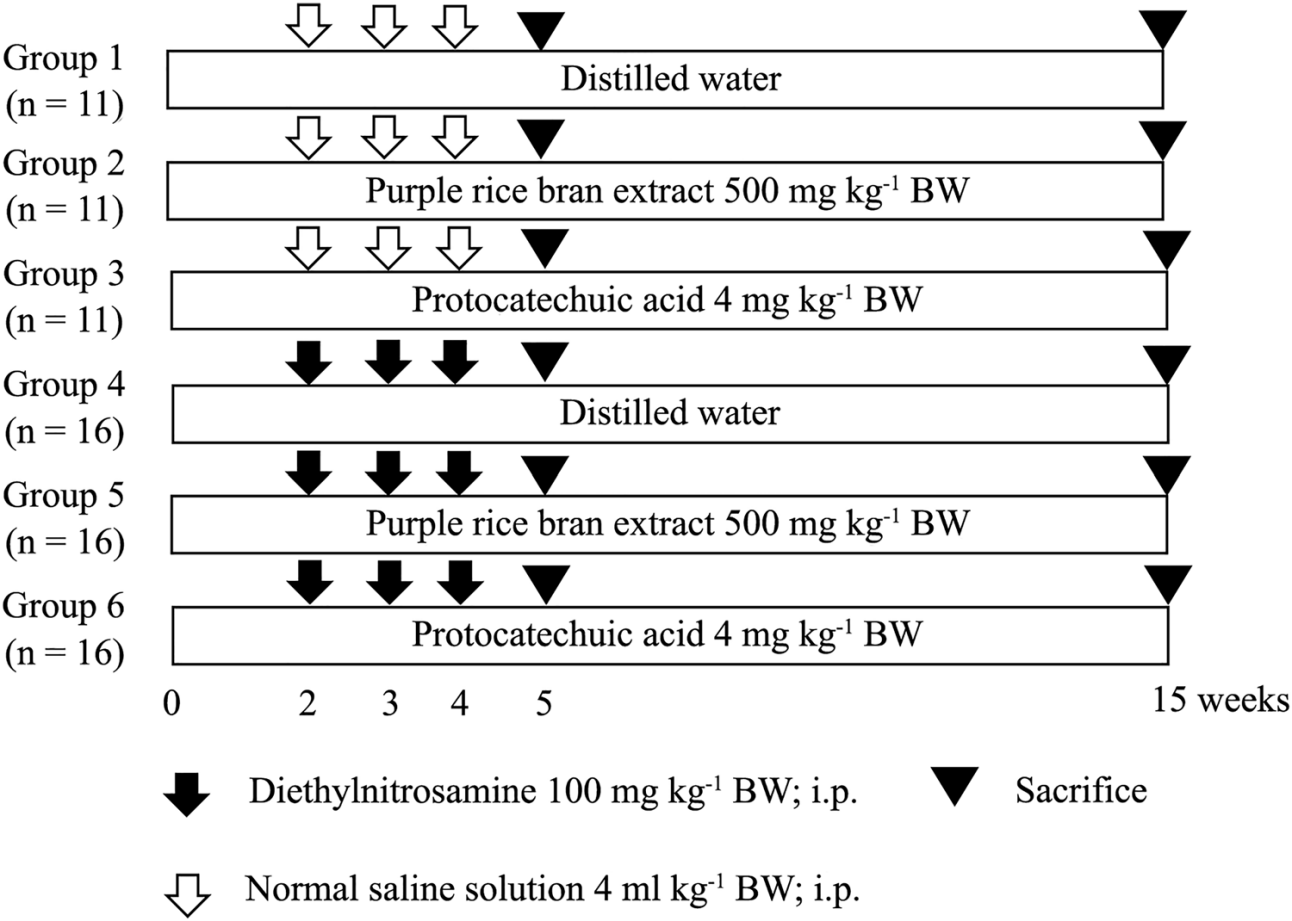
Experimental protocol for testing the anticarcinogenic effect of purple rice bran extract and protocatechuic acid.

To determine the chemopreventive effect of PRBE and PCA on DEN-induced rat hepatocarcinogenesis, male Wistar rats (4 weeks old, 90–110 g) were divided into six groups. Rats in groups 1–3 were injected intraperitoneally with normal saline, while groups 4–6 were injected with 100 mg kg^−1^ BW of DEN in weeks 2, 3, and 4 of an experiment to initiate hepatocarcinogenesis. Group 1 was a negative control, while group 4 was a positive control. Rats in group 2 were fed with 500 mg kg^−1^ BW of PRBE via intragastric gavage feeding, whereas group 3 was fed with 4 mg kg^−1^ BW of PCA, to study their carcinogenicity. Two weeks before DEN injection, rats in group 5 were fed with 500 mg kg^−1^ BW of PRBE, while group 6 orally received PCA at a concentration of 4 mg kg^−1^ BW, to evaluate its anticancer activity. All rats were dosed once daily for 5 days per week. In week 5 of the experiment, six rats per group were sacrificed to determine the effect of PRBE and PCA on the initiation stage of hepatocarcinogenesis. The remaining rats were sacrificed in week 15 of an experiment to evaluate the effect of PRBE and PCA on the promotion stage of hepatocarcinogenesis. Body weight, water, and food intake were measured twice a week throughout the experiment. The weights of major organs including liver, kidney, and spleen were measured to evaluate the toxic effect of test compounds for the 5- and 15-week protocols. The treatment protocol is shown in Fig. 1.

### Determination of glutathione *S*-transferase placental form positive foci in rat liver (5- and 15-week protocols)

To investigate the effect of PRBE and PCA on the formation of hepatic preneoplastic lesions, the GST-P positive foci were stained by immunohistochemistry according to Thumvijit et al.^15^. Briefly, liver sections were deparaffinized with xylene and dehydrated with ethanol. Endogenous peroxidase activity and non-specific protein bindings were blocked with H_2_O_2_ and nonfat milk, respectively. The slides were treated with an anti-rabbit GST-P antibody (MBL, Japan) diluted 1:1000. Then, they were incubated with a secondary antibody conjugated to an avidin–biotin peroxidase complex (Vector Laboratories, Inc., USA). The GST-P positive cells were detected using 3,3′-diaminobenzidine as a substrate. The number and area of GST-P-positive foci larger than 0.15 mm^2^ and 0.2 mm^2^ were measured in the 5- and 15-week experiments, respectively, using the Leica Application Suite (LAS) Interactive Measurement program (Leica Microsystems, Germany).

### Evaluation of some phase I and II xenobiotic-metabolizing enzymes (5-week protocol)

To study the effect of PCA on the initiation stage of rat hepatocarcinogenesis, the activity and expression of some phase I and II xenobiotic-metabolizing enzymes in rat livers were investigated. The liver cytosolic and microsomal fractions were prepared according to Punvittayagul et al. (2011) and the protein concentration was determined by the Lowry method. The activity of cytochrome P450 reductase (CPR), UDP-glucuronyltransferase (UGT), and GST was determined according to our previous report^16^.

The expression of cytochrome P450 2E1 was determined by Western blot analysis using polyclonal anti-rat CYP2E1 antibody (Thermo Fisher Scientific, USA) at 1:12,000 dilution. The protocol was performed according to Punvittayagul et al. (2011)^16^. Protein detection was performed using HRP-conjugated secondary antibodies (Abcam, UK) and an enhanced chemiluminescent kit (Thermo Fisher Scientific, USA). The intensity of each band was evaluated with the Image J program, which was normalized against protein disulfide isomerase (PDI, Cell Signaling Technology, USA).

### Assessment of proliferating cell nuclear antigen and apoptotic hepatocytes by double-staining immunohistochemistry (15-week protocol)

To evaluate the effect of PRBE and PCA on cell proliferation and apoptosis, double-staining procedures were carried out using an EnVision Doublestain system from Dako (Hamburg, Germany). Liver sections were stained with anti-PCNA antibody (Biolegend, USA) at 1 : 2000 dilution and anti-GST-P antibody (MBL, Japan) at 1 : 1000 dilution, following the manufacturer’s instructions. The number of PCNA-positive cells was counted under a light microscope as described in our previous study^8^.

The terminal deoxynucleotidyl transferase dUTP nick end labeling (TUNEL) assay was used to identify apoptotic hepatocytes. A double-labeling assay for TUNEL and GST-P was carried out using an ApopTag Peroxidase in situ kit (Merck, Germany) and EnVision Doublestain system (Dako, Denmark) according to Thumvijit et al. (2014)^15^. The number of apoptotic labeled cells was recorded as in our previous reports^8^.

### Gene expression analysis by real-time polymerase chain reaction (5- and 15-week protocols)

The effect of PCA on the mRNA levels of genes involved in the initiation and promotion stages of hepatocarcinogenesis was determined by real-time polymerase chain reaction (RT-PCR). The liver tissue samples were homogenized in PureZOL™ RNA Isolation Reagent (Bio-Rad, USA), following the manufacturer’s procedure. cDNAs were synthesized using a High-Capacity cDNA Reverse Transcription Kit (Applied Biosystems™, USA), according to the supplier’s protocol. Quantitative RT-PCR was carried out using specific primers (Integrated DNA Technologies, Inc., Singapore) as listed in Table 1. All PCR reactions were performed using a SensiFast™ SYBR Lo-ROX Kit (Bioline, France) and were carried out under the following conditions: initial denaturation at 95 °C for 1 min followed by 40 cycles of 15 s at 95 °C, 15 s at 56–60 °C and 10 s at 72 °C. Differences in gene expression between groups were calculated by the 2^−ΔΔCT^ method, which was normalized against β-actin and expressed as relative expression compared with the control.

**Table 1:** Primer lists for the real-time polymerase chain reaction.

Gene	5′-3′ Primer sequence	References
*TNF-α*	Forward: 5′-AAA TGG CCC TCT CAT CAG TCC-3′ Reverse: 5′-TCT GCT TGG TGG TTT GCT ACG AC-3′	^14^
*IL-1β*	Forward: 5′-CAC CTC TCA AGC AGA GCA CAG-3′ Reverse: 5′-GGG TTC CAT GGT GAA GTC AAC-3′	^14^
*Cyclin D1*	Forward: 5′-GTC GAG AAG AGA AAG CTC TG-3′ Reverse: 5′-TTA AAA GCC TCC TGT GTG AA-3′	^17^
*P53*	Forward: 5′-CTT CGA GAT GTT CCG AGA GC-3′ Reverse: 5′-CTT CGG GTA GCT GGA GTG AG-3′	^18^
*Bad*	Forward: 5′-GGA GCA TCG TTC AGC AGC AG-3′ Reverse: 5′-CCA TCC CTT CAT CTT CCT CAG TC-3′	^19^
*Bcl-xl*	Forward: 5′-AGG CTG GCG ATG AGT TTG AA-3′ Reverse: 5′-TGA AAC GCT CCT GGC CTT TC-3′	^20^
*β-Actin*	Forward: 5′-ACA GGA TGC AGA AGG AGA TTA C-3′ Reverse: 5′-AGA GTG AGG CCA GGA TAG A-3′	^14^

### Statistical analysis

Statistical comparison between groups was conducted using a one-way analysis of variance and the least significant difference (LSD) method was used to determine significantly different groups. Statistical significance was accepted as *p* ≤ 0.05.

## Results

### Anticarcinogenic effect of purple rice bran extract and protocatechuic acid against diethylnitrosamine-induced rat hepatocarcinogenesis

After 5 weeks of the experiment, final body weight was significantly reduced in DEN-initiated rats compared to the control group. This result was directly related to the amount of food and water consumed. This result indicates that the DEN treatment was toxic to the rats. However, treatment with PRBE and PCA slightly improved the body weight when compared with the positive control group. By the end of week 15 of the experiment, there was no significant difference in body weight or food or water consumption among groups. There was no considerable alteration in the body weight or food or water consumption of rats administered PRBE and PCA alone for 5 and 15 weeks (data not shown). These findings indicate that PRBE and PCA had no adverse effect on the growth of the rats.

One week after the last DEN injection (5-week protocol), the liver weight of DEN-treated rats decreased significantly compared to the negative control animals. Remarkably, PRBE and PCA treatment significantly improved liver weight in DEN-initiated rats. However, there were no significant intergroup differences in the relative weights of the kidney and spleen. At the time point of 15 weeks, there was no significant difference in kidney, spleen, or liver weights between groups. The results are presented in Table 2. These findings indicated that PRBE and PCA did not present any toxicity when administered to rats.

**Table 2:** Relative organ weight of rats with DEN-induced hepatocarcinogenesis treated with purple rice bran extract and protocatechuic acid.

Treatment	Initiation (5-week protocol)			Promotion (15-week protocol)		
	Relative organ weight			Relative organ weight		
	Liver	Spleen	Kidney	Liver	Spleen	Kidney
NSS	4.02 ± 0.21	0.66 ± 0.03	0.23 ± 0.04	3.16 ± 0.23	0.23 ± 0.04	0.51 ± 0.02
NSS + PRBE 500 mg kg^−1^ BW	4.12 ± 0.18	0.68 ± 0.04	0.20 ± 0.02	2.94 ± 0.25	0.17 ± 0.03	0.49 ± 0.04
NSS + PCA 4 mg kg^−1^ BW	4.32 ± 0.05	0.68 ± 0.03	0.24 ± 0.03	3.12 ± 0.19	0.18 ± 0.01	0.47 ± 0.03
DEN	3.08 ± 0.14*	0.66 ± 0.03	0.28 ± 0.04	3.06 ± 0.22	0.19 ± 0.02	0.53 ± 0.04
DEN + PRBE 500 mg kg^−1^ BW	3.36 ± 0.11^#^	0.68 ± 0.03	0.26 ± 0.03	3.12 ± 0.26	0.19 ± 0.02	0.53 ± 0.02
DEN + PCA 4 mg kg^−1^ BW	3.50 ± 0.18^#^	0.70 ± 0.03	0.29 ± 0.03	2.92 ± 0.10	0.19 ± 0.01	0.51 ± 0.02

Values are expressed as mean ± SD.

DEN: diethylnitrosamine, NSS: normal saline solution, PCA: protocatechuic acid, PRBE: purple rice bran extract.

*Significantly different from the negative control group, *p* < 0.05.

^#^Significantly different from the positive control group, *p* < 0.05.

**Figure 2:**
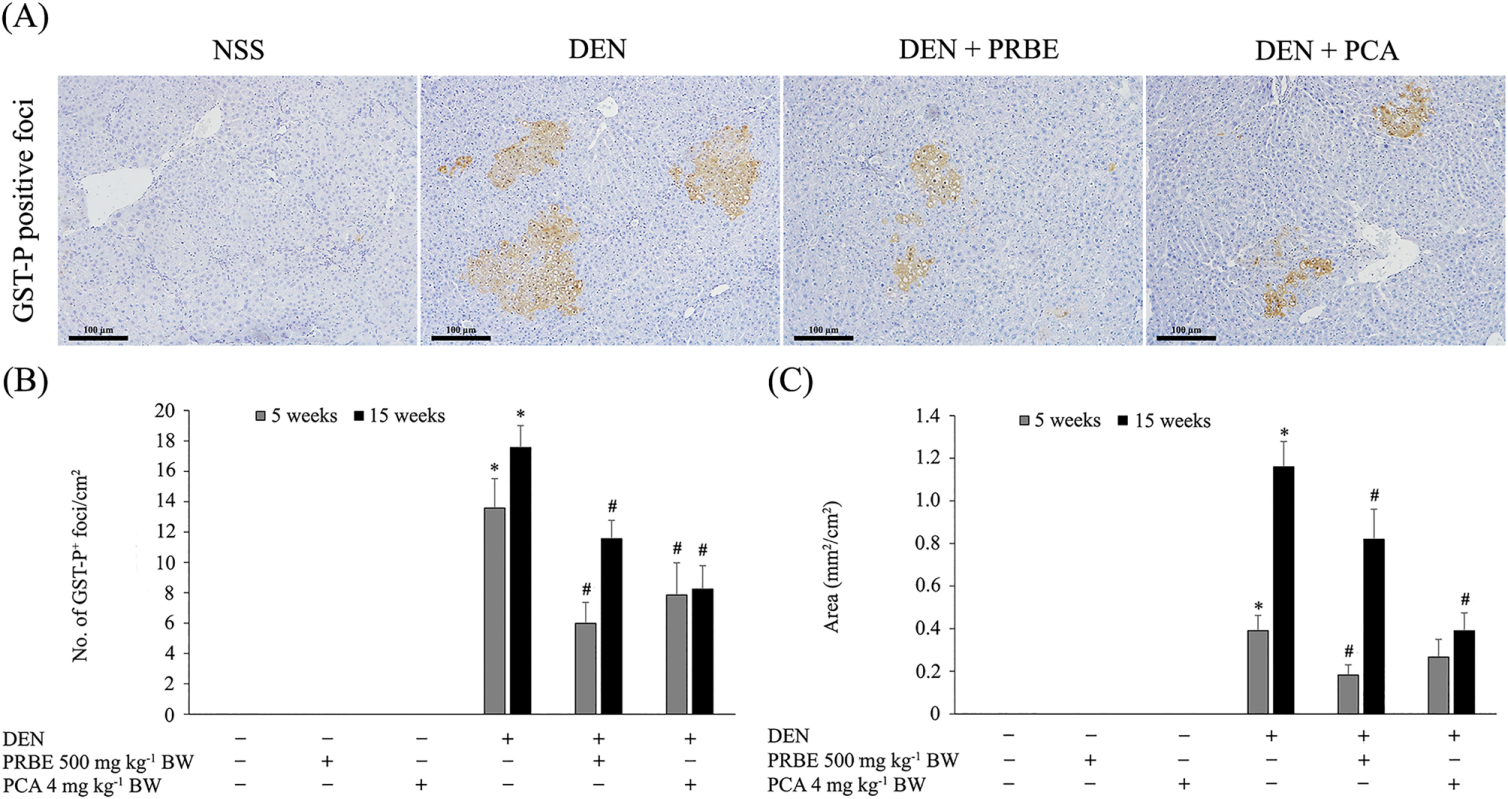
Effect of purple rice bran extract and protocatechuic acid on the number and area of hepatic GST-P-positive foci. (**A**) Immunohistochemical staining of GST-P-positive foci (brown) (20 ×); (**B**) number of GST-P^+^ foci/cm^2^; (**C**) area (mm^2^/cm^2^). Values are expressed as mean ± SEM. *Significantly different from the negative control group, *p* < 0.05. ^#^Significantly different from the positive control group, *p* < 0.05. DEN: diethylnitrosamine, PCA: protocatechuic acid, PRBE: purple rice bran extract.

Figure 2 illustrates the effect of PRBE and PCA on the number and area of GST-P-positive foci. By the end of weeks 5 and 15 of the experiments, no GST-P formation was detected in the groups given PRBE and PCA alone, suggesting a lack of carcinogenicity. A significant increase in the number and area of GST-P-positive foci was observed in the DEN-treated group compared to their control group. Interestingly, PRBE and PCA treatments in DEN-induced rats resulted in a significant reduction of GST-P-positive foci compared to the group given DEN alone. These results indicate that PRBE and PCA had preventive effects against hepatocarcinogenesis caused by DEN treatment. Comparatively, the levels of anticarcinogenic activity of PCA and PRBE were similar to each other.

### Effect of protocatechuic acid on some phase I and phase II xenobiotic-metabolizing enzymes

**Figure 3:**
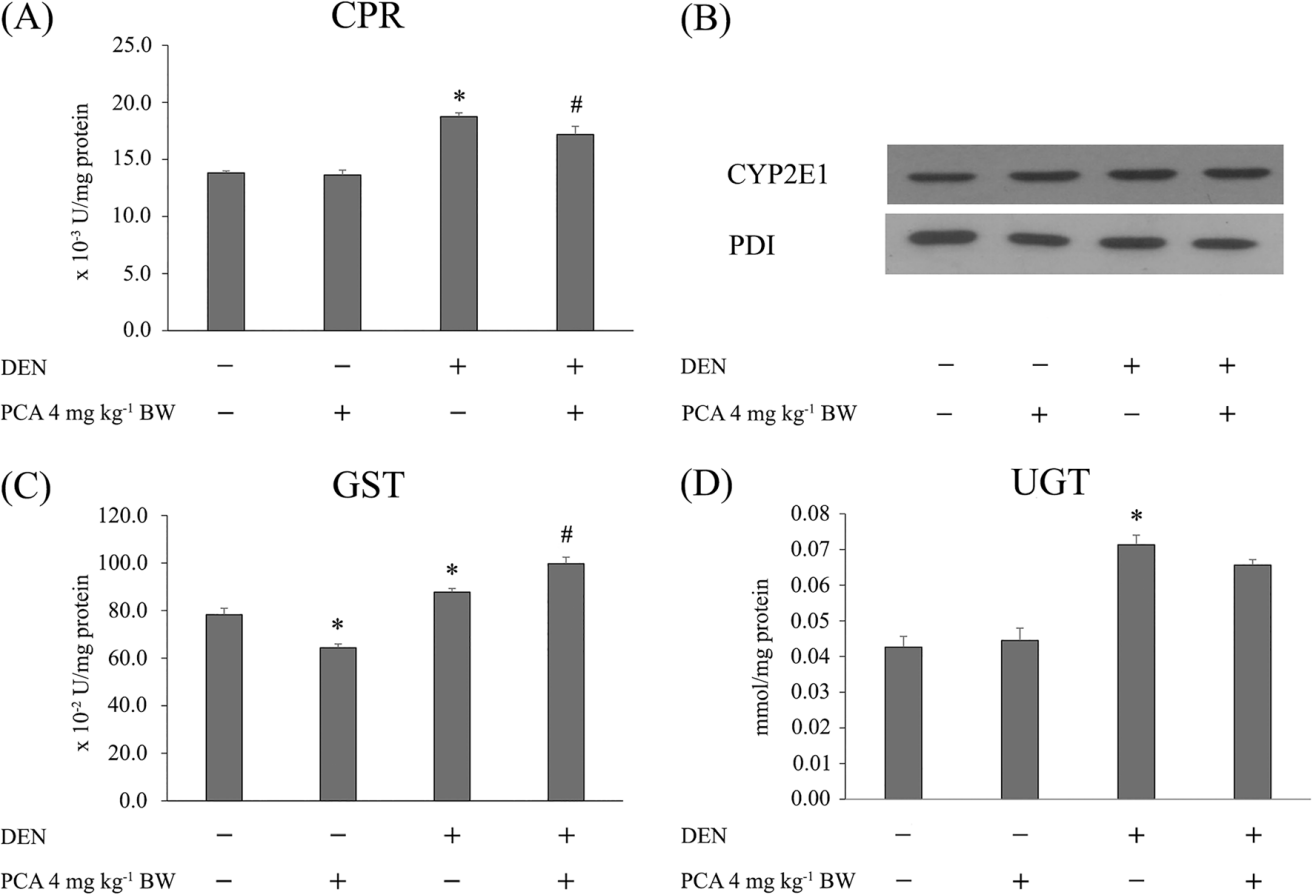
Effect of purple rice bran extract and protocatechuic acid on some phase I and phase II xenobiotic-metabolizing enzymes (5-week protocol). (**A**) CPR: NADPH: cytochrome P450 reductase activity; (**B**) Western blotting of cytochrome P450 2E1 (CYP2E1); (**C**) GST: glutathione-*S*-transferase activity; (**D**) UGT: UDP-glucuronyltransferase. Values are expressed as mean ± SEM. *Significantly different from the negative control group, *p* < 0.05. ^#^Significantly different from the positive control group, *p* < 0.05. DEN: diethylnitrosamine, PCA: protocatechuic acid, PRBE: purple rice bran extract.

Protection against carcinogen-induced carcinogenesis can be accomplished by phase I inhibition and phase II induction. Figure 3 illustrates the activity and expression of some phase I and phase II xenobiotic-metabolizing enzymes in rat liver. Administration of DEN significantly increased the activity of CPR, an electron donor, when compared with the control. Remarkably, supplementation of the DEN-treated rats with PCA significantly decreased CPR activity, whereas treatment with PCA alone did not result in any significant variation from the negative control group (Fig. 3A). In addition, PCA treatment did not lead to any alteration in the expression of CYP2E1, a key enzyme in DEN metabolism (Fig. 3B). The activity of UGT and GST was significantly enhanced in DEN-administered rats. PCA-treated rats displayed a significant increase in GST activity as compared to DEN-treated rats but did not show any effect on UGT activity. However, treatment with PCA alone decreased GST activity (Fig. 3C and D). These results indicate that PCA acts as a dual-acting agent by reducing phase I enzyme activity and inducing phase II enzyme activity, thereby promoting detoxification and excretion.

### The alteration of cell proliferation and apoptosis of purple rice bran extract and protocatechuic acid in rats

**Figure 4:**
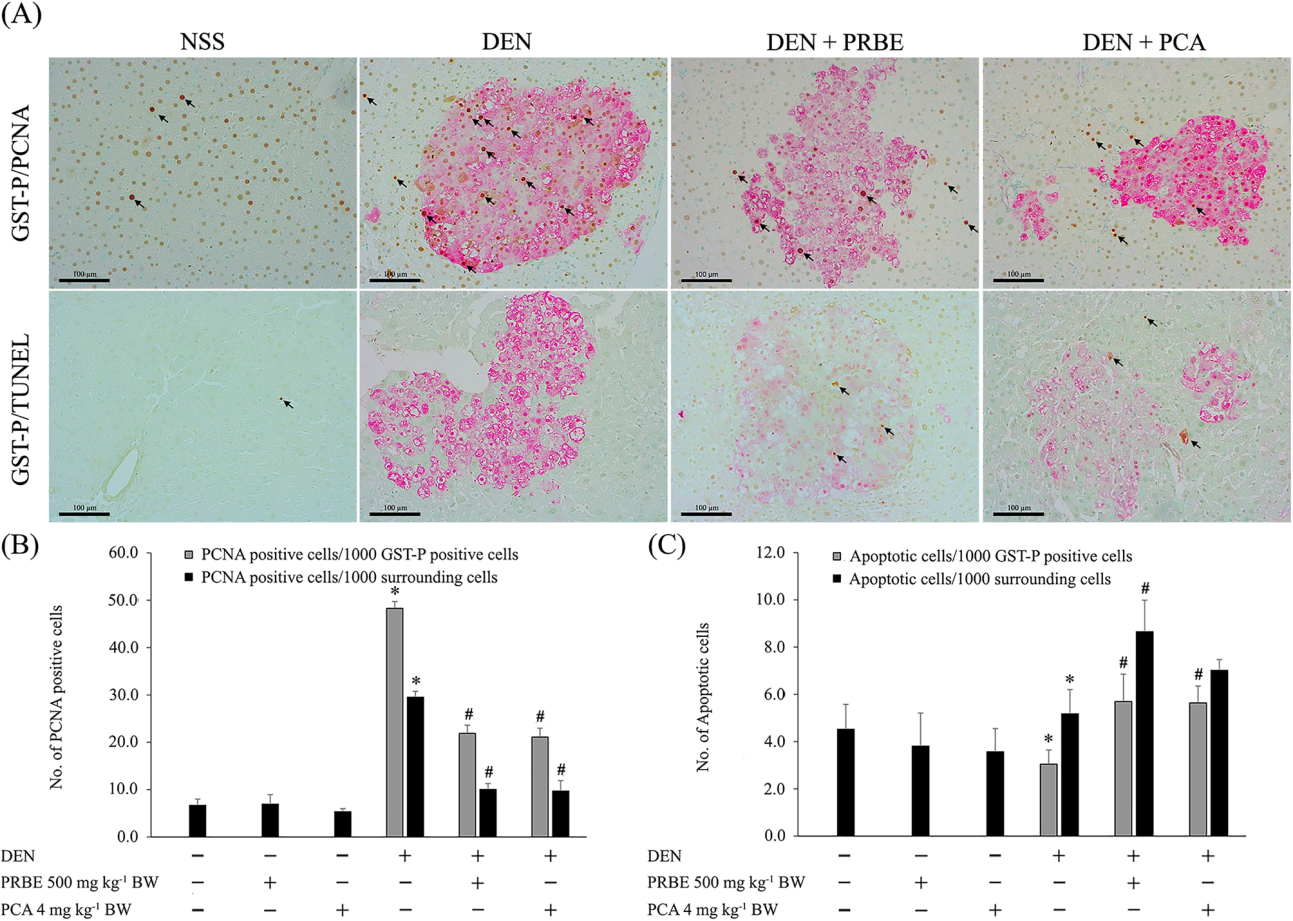
Effect of purple rice bran extract and protocatechuic acid on cell proliferation and apoptosis (15-week protocol). Arrowheads indicate stained hepatocytes. (**A**) Representative double immunohistochemical staining of GST-P (red)/PCNA (brown) and GST-P (red)/apoptosis (TUNEL) (black) in the liver of control and experimental animals (20 ×); (**B**) number of PCNA-labeled cells per 1000 hepatocytes; (**C**) number of apoptotic cells per 1000 hepatocytes. Values are expressed as mean ± SEM. *Significantly different from the negative control group, *p* < 0.05. ^#^Significantly different from the positive control group, *p* < 0.05. DEN: diethylnitrosamine, PCA: protocatechuic acid, PRBE: purple rice bran extract.

Figure 4 shows the effect of PRBE and PCA on cell proliferation and apoptosis in rat livers. Double immunohistochemical staining of GST-P/PCNA and GST-P/apoptotic cells is shown in Fig. 4A. PCNA is a nuclear protein, widely used as a standard marker of cell proliferation, which was found to be significantly increased in DEN-treated rats. Administration of PCA at the tested dose level significantly decreased the number of PCNA-positive cells in both GST-P-positive foci and the surrounding area in DEN-initiated rats. In addition, PCA significantly increased apoptosis in GST-P-positive foci of DEN-induced rats, but not in the surrounding area (Fig. 4B and C). These results produced by PCA were almost comparable with those for PRBE. These findings indicate that PCA suppresses the development of liver cancer by reducing cell proliferation and inducing cell apoptosis in DEN-initiated rats.

### Response of protocatechuic acid to pro-inflammatory, cell proliferation, and apoptosis-related gene expression in diethylnitrosamine-initiated rats

**Figure 5:**
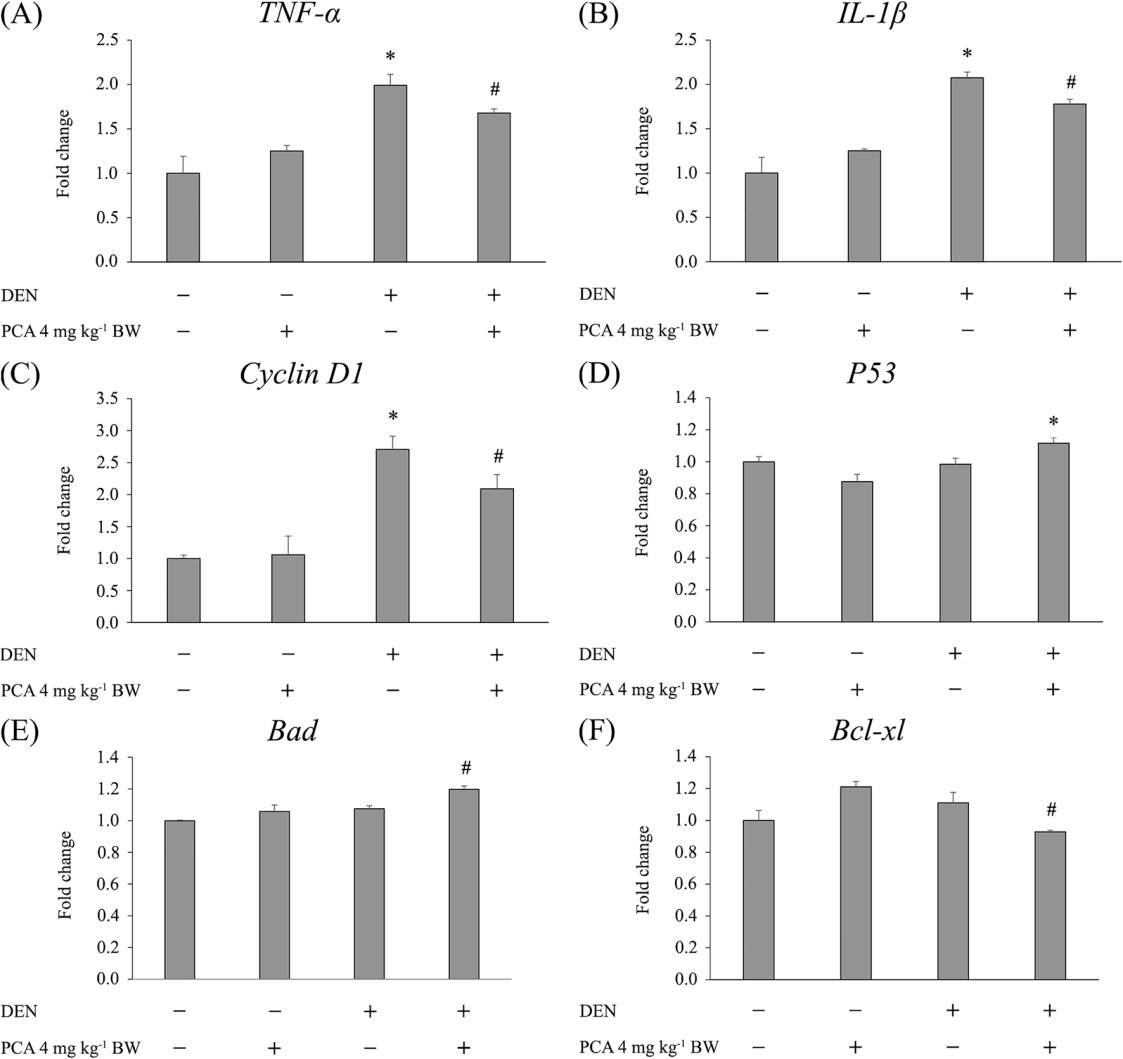
Effect of protocatechuic acid on mRNA levels of genes involved in the initiation and promotion stages of hepatocarcinogenesis (5- and 15-week protocols). Data were normalized to the *β-actin* mRNA level and are expressed as the fold difference versus the negative control group. (**A**) *TNF-α*; (**B**) *IL-1β*; (**C**) *Cyclin D1*; (**D**) *P53*; (**E**) *Bad*; (**F**) *Bcl-xl*. Values are expressed as mean ± SEM. *Significantly different from the negative control group, *p* < 0.05. ^#^Significantly different from the positive control group, *p* < 0.05. DEN: diethylnitrosamine, PCA: protocatechuic acid.

The effect of PCA on genes associated with inflammation, cell proliferation, and apoptosis is presented in Fig. 5. After 5 weeks of the experiment (initiation stage), DEN-treated animals demonstrated significant upregulation of *TNF-α* and *IL-1β* mRNA in the liver compared to control animals. Oral administration of PCA significantly downregulated the expression of *TNF-α* and *IL-1β* mRNA in DEN-initiated rats when compared to the group given DEN alone (Fig. 5A and B). After 15 weeks of the experiment (promotion stage), a significant increase in the mRNA expression of *Cyclin D1*, a key regulator of G1/S phase transition, was observed in the DEN-treated group compared to the negative control group. Interestingly, supplementation with PCA significantly decreased the expression of *Cyclin D1* when compared to the DEN-treated group (Fig. 5C). Furthermore, administration of PCA to DEN-treated rats significantly increased the expression of *P53* and *Bad* (pro-apoptotic genes) accompanied by a decrease of *Bcl-xl* (anti-apoptotic gene) expression (Fig. 5D–F). In comparison, the control and PCA-alone groups presented similar results. Our findings demonstrate that PCA has potent anti-inflammatory action against the DEN-induced initiation stage of hepatocarcinogenesis by reducing the expression of the pro-inflammatory cytokines *TNF-α* and *IL-1β*. In the promotion stage, the anti-cell proliferative effect has been linked to the decrease of *Cyclin D1*. It also efficiently triggered apoptosis by induction of *P53* and the pro-apoptotic gene *Bad*, accompanied by reduction of the anti-apoptotic gene *Bcl-xl*.

## Discussion

Food is not only a source of energy but also contains several bioactive compounds that have been shown to possess health-promoting properties^4,5^. Rice is the chief food for more than half of the world’s population. Our recent experiment demonstrated the effectiveness of PRBE against DEN-induced rat hepatocarcinogenesis^14^. In this study, PCA, the most abundant phenolic acid in PRBE, could inhibit DEN-induced formation of GST-P positive foci similarly to PRBE. Previous reports have shown the cancer protective activities of PCA, such as prevention of carcinogen biotransformation, detoxification of xenobiotics, anti-inflammation, reduction of cell proliferation, and induction of apoptosis in several types of cancers^21–23^. Yip et al.^24^ have demonstrated that PCA could induce cell death in HepG2 hepatocellular carcinoma cells. It also inhibits rat hepatocarcinogenesis induced by DEN^25^. In addition, nanoparticles containing protocatechuic acid suppressed liver tumors in DEN- and phenobarbital-induced mice^26^. Although the anticarcinogenic effects of PCA on DEN-initiated liver cancer have been reported, they lacked the relevant amount of PCA in food. This study first showed that PCA at the dose of 4 mg kg^−1^ body weight attenuated DEN-induced early stages of rat hepatocarcinogenesis with the efficacy as PRBE. It might be indicated that PCA is an anticarcinogenic compound of PRBE. In addition, these findings contribute to understanding the underlying molecular mechanisms by which PCA ameliorates DEN-induced hepatocarcinogenesis.

The biotransformation of DEN by the cytochrome P450–dependent pathway produces active metabolites, which are primarily responsible for the initiation of hepatocarcinogenesis^27^. However, the electrophilic intermediates can be eliminated by making conjugates with hydrophilic molecules such as glutathione and glucuronic acid^28^. Previous investigation has shown that PCA reduced the activity and expression of CYP1A1/2 and CYP2E1 but induced the activity of GST and NQO1 in 3-methylcholantren-induced rats^29^. Recently, our report has shown that PRBE does not affect the activity of phase I and phase II enzymes^14^. Interestingly, the decreased activity of CPR accompanied by increased activity of GST observed in the present study provides evidence for the preventive action of PCA in DEN-induced rat hepatocarcinogenesis. Our results demonstrate that PCA acts as a potential dual-acting agent by inhibiting phase I enzymes and activating phase II enzymes. Other than that, inflammation is a critical factor contributing to the development of cancer. Interleukin 1 (IL-1) and tumor necrosis factor α (TNF-α) are considered key molecules for cancer progression^30^. The increased levels of *IL-1β* and *TNF-α* presented in this study provide evidence that inflammation was induced in DEN-treated rats. Importantly, PCA can downregulate the expression of *IL-1β* and *TNF-α* genes in DEN-initiated rats, presenting the same effect as PRBE^14^. This result is supported by a previous report that PCA suppresses the production of IL-1β and TNF-α in LPS-stimulated RAW 264.7 cells and also inhibits COX-2 expression in carrageenan-induced inflammation in mice^31^. These findings revealed that PCA inhibits liver inflammation, resulting in the suppression of the development of hepatocarcinogenesis.

The progression of cancer is related to an increase in the proportion of proliferating cells. One common approach to studying cell proliferation activity is immunohistochemical staining of PCNA, which is a marker of cell transformation^32^. DEN-initiated rats showed an increased number of PCNA, indicating hyperproliferative activity. However, PCA treatment in DEN-treated rats decreased the level of PCNA in both hepatic GST-P-positive foci and the surrounding area. This effect revealed that PCA attenuates DEN-induced hepatocarcinogenesis by suppressing cell proliferation. This positive result is consistent with our previous study in which PRBE inhibited cell proliferation in DEN-treated rats^14^. Therefore, it may be assumed that PCA acts as a potent inhibitor of cancer cell proliferation in PRBE. Overexpression of cyclin D1 has been linked to the development of cancers because it is an essential regulator that activates the transition from G1 to S phase^33,34^. DEN treatment upregulated the expression of *Cyclin D1*, whereas PCA downregulated the expression of the *Cyclin D1* gene in DEN-administered rats. This finding demonstrates that PCA suppresses cell proliferation via downregulation of *Cyclin D1* in DEN-induced rat hepatocarcinogenesis.

Apoptosis is an essential physiological process for cell homeostasis. The inhibition of apoptosis contributes to the pathogenesis of cancer^35^. Alteration of p53, a tumor suppressor gene, is one of the key regulators in cell cycle arrest and apoptosis. Normally, DNA-damaged cells undergo apoptosis which is a protective mechanism to prevent the development of cancer^36^. PCA administration increased the number of TUNEL-positive cells in the DEN-treated group. This finding was similar to that observed for the activity of PRBE. Additionally, treatment with PCA increased the level of *P53*, thereby potentially driving the mutated cells to apoptosis. A previous report demonstrated that the Bcl-2 antagonist of cell death (BAD)-mediated apoptotic pathway plays an important role in carcinogenesis. To initiate apoptosis, Bad protein dimerizes with Bcl-xL and Bcl-2, displacing Bax, leading to the induction of cell apoptosis^37^. Previous investigation has reported that Bad is transcriptionally upregulated by p53 and it can also form a Bad/p53 complex at the mitochondria to induce apoptosis^38^. In this study, the quantitative results showed that PCA induced programmed cell death in DEN-treated rats through upregulation of *Bad* (a pro-apoptotic gene) and downregulation of *Bcl-xl* (an anti-apoptotic gene). Taken together, these data suggest that PCA induces apoptosis via a mitochondria-mediated apoptotic pathway.

This study demonstrates for the first time that PCA (4 mg kg^−1^ BW) is an active compound in the anticarcinogenic action of purple rice bran against the formation of DEN-induced hepatic preneoplastic lesions in rats. The preventive mechanisms were associated with xenobiotic-metabolizing enzymes (decreased CPR and increased GST enzyme activity). Additionally, PCA also exerted anti-inflammatory activity (downregulating mRNA levels of the pro-inflammatory cytokines *TNF-α* and *IL-1β*). Moreover, it suppressed hepatic cell proliferation by decreasing *Cyclin D1*. PCA also induced hepatic apoptosis via upregulation of *P53* and *Bad* and downregulation of *Bcl-xl* mRNA levels. Notably, the effective dose of PCA, 4 mg kg^−1^ BW, corresponds to a human-equivalent dose (HED) of 39 mg/day^39^, which is greater than the daily recommended dose of PCA in food, approximately 2–10 mg g^−1^ ^21^. However, the cancer chemopreventive dose of PCA is only estimated within a critical period of carcinogen exposure. PCA is one of the active metabolites of dietary anthocyanins^40^; therefore, the regular consumption of anthocyanin-rich foods such as purple rice and other colored vegetables and fruits should be recommended. This knowledge might provide an important implication for the use of PCA as a potential anticancer agent in humans. Nevertheless, clinical trials testing this compound need to be performed.

## Supplementary Information

Supplementary Information 1.

Supplementary Information 2.

## Data Availability

The datasets generated during and analyzed during the current study are available from the corresponding author on reasonable request.
