# Supplementary figures and images for: Protocatechuic acid as a potent anticarcinogenic compound in purple rice bran against diethylnitrosamine-initiated rat hepatocarcinogenesis
Source: Sci Rep. 2022 Jun 22;12:10548. doi: 10.1038/s41598-022-14888-2 (PMC9217852; doi:10.1038/s41598-022-14888-2)

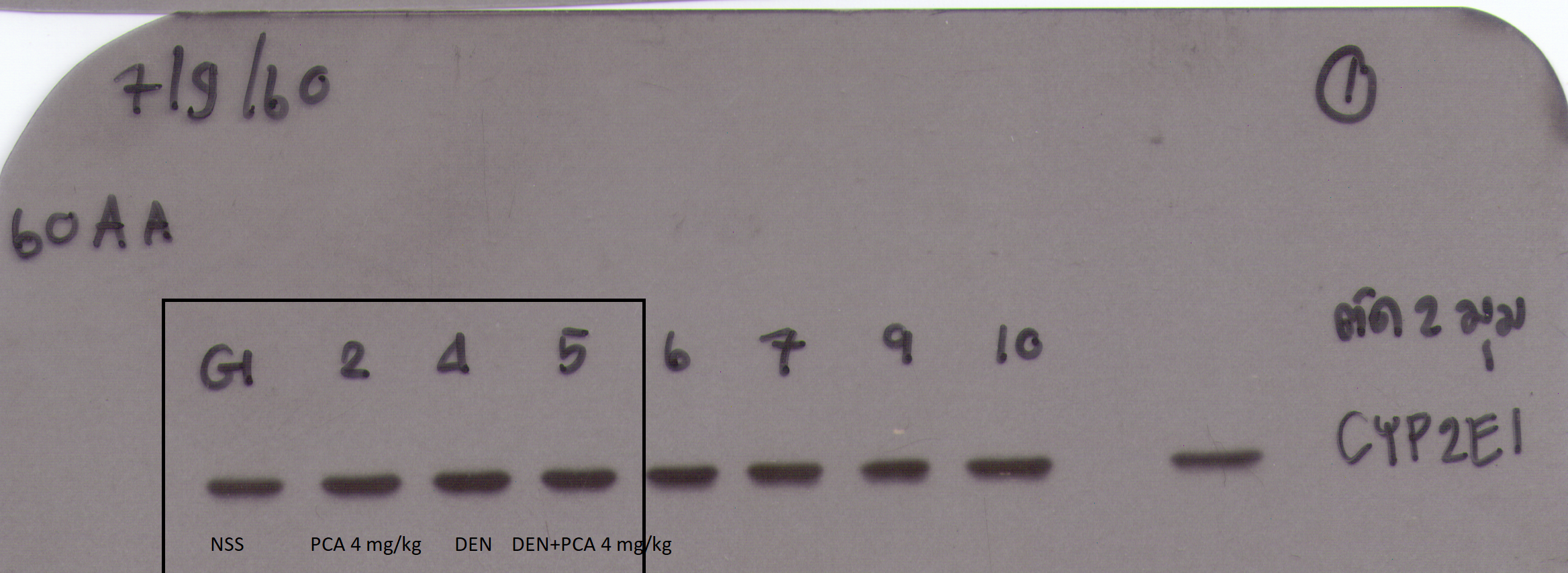

Supplement: Supplementary file 1 — Supplementary Information 1. [file 41598_2022_14888_MOESM1_ESM.tif]

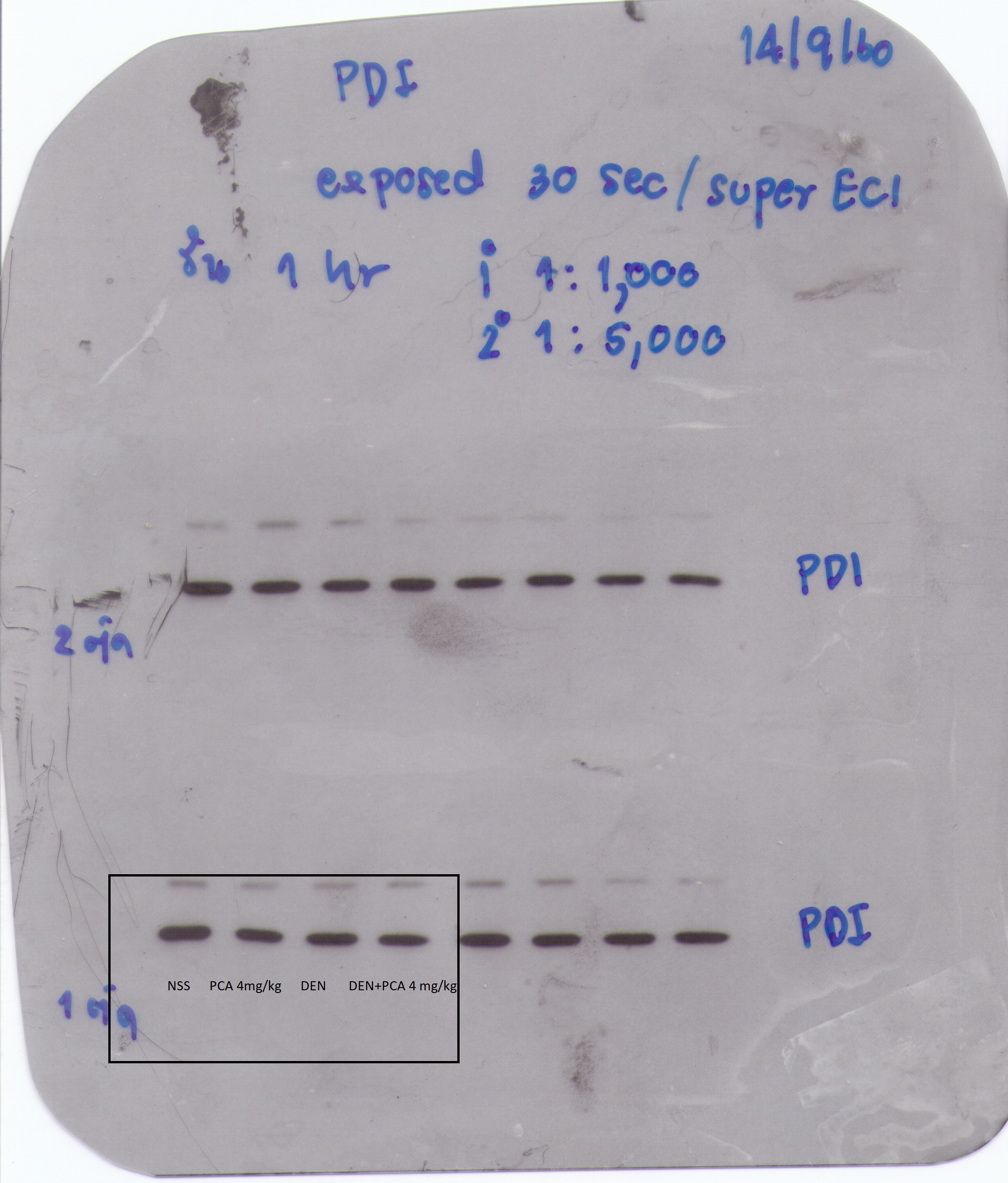

Supplement: Supplementary file 2 — Supplementary Information 2. [file 41598_2022_14888_MOESM2_ESM.tif]
